# DNAJB4 promotes triple-negative breast cancer cell apoptosis via activation of the Hippo signaling pathway

**DOI:** 10.1007/s12672-023-00645-y

**Published:** 2023-04-04

**Authors:** Fang Fang, Linglong Mo, Xiaofeng Pan, Ziquan Yang, Haoyu Huang, Liangyu Zhu, Yingying Wang, Guoqin Jiang

**Affiliations:** 1 Department of Surgery, The Second Affiliated Hospital of Soochow University, 1055 San-Xiang Road, Suzhou, 215004 China; 2 Department of Surgery, The First Affiliated Hospital of Wannan Medical College, Yijishan Hospital of Wannan Medical College, Wuhu, 241001 China; 3 Key Laboratory of Non-Coding RNA Transformation Research of Anhui Higher Education Institutes, Wannan Medical College, Wuhu, 241001 China; 4 Department of Nuclear Medicine, The First Affiliated Hospital of Wannan Medical College, Yijishan Hospital of Wannan Medical College, Wuhu, 241001 China

**Keywords:** DNAJB4, TNBC, Cell apoptosis, Hippo signaling pathways, Biomarker

## Abstract

**Introduction:**

Triple-negative breast cancer (TNBC) is currently the most malignant subtype of breast cancer without effective targeted therapies. DNAJB4 (Dnaj heat shock protein family (Hsp40) member B4) is a member of the human heat shock protein family (Hsp40). The clinical significance of DNAJB4 in breast cancer has been reported in our previous study. However, the biological function of DNAJB4 in TNBC cell apoptosis remains unclear to date.

**Methods:**

The expression of DNAJB4 in normal breast cells, breast cancer cells, four-paired TNBC tissues, and adjacent noncancerous tissues was quantified by quantitative real-time polymerase chain reaction (qRT-PCR) and western blot assay. The role of DNAJB4 in TNBC cell apoptosis was investigated using a number of gain- and loss-of-function in vitro and in vivo assays. The underlying molecular mechanisms in TNBC cell apoptosis were elucidated via Western blot assay.

**Results:**

DNAJB4 expression was significantly downregulated in TNBC tissues and cell lines. DNAJB4 knockdown inhibited TNBC cell apoptosis and promoted tumorigenicity in vitro and in vivo, but DNAJB4 overexpression resulted in the opposite. Mechanically, DNAJB4 knockdown inhibited TNBC cell apoptosis through suppression of the Hippo signaling pathway, and the result was reversed after DNAJB4 overexpression.

**Conclusions:**

DNAJB4 promotes TNBC cell apoptosis by activating the Hippo signaling pathway. Therefore, DNAJB4 may act as a prognostic biomarker and therapeutic target for TNBC.

**Supplementary Information:**

The online version contains supplementary material available at 10.1007/s12672-023-00645-y.

## Introduction

According to the latest global cancer data for 2020 released by the World Health Organization’s International Agency for Research on Cancer (IARC), breast cancer (BC), whose incidence exceeds that of lung cancer in the general population, ranks first among malignant tumors and fifth in terms of mortality [1]. According to Chinese epidemiological statistics in 2015, there are approximately 1.38 million new breast cancer cases and 450,000 deaths in China every year, and its incidence is still increasing yearly and has a younger trend [2]. Triple-negative breast cancer (TNBC) is currently the most malignant subtype of breast cancer without effective targeted therapies [3]. Currently, the conventional treatment for TNBC is surgery, chemotherapy, radiotherapy, endocrine therapy, and targeted therapy, combined with other adjuvant therapy. Although standard treatment is effective for early TNBC, some breast cancer patients still experience recurrence and metastasis, and there is currently no effective treatment for this situation. According to statistics, the main cause of death in TNBC patients is TNBC metastasis to distant organs or related complications caused by metastasis, thereby making its pathogenesis an important target for research [4].

Apoptosis is cell death caused by the activation of intracellular energy-dependent death programs. It is an autonomous and orderly active cell death process controlled by genes [5]. Under normal circumstances, apoptosis has a protective effect on the body, being a death process that is actively adopted to better adapt to the living environment [6]. Disturbance of this process can lead to various diseases, such as cancer [7]. Increasing evidence indicates that inhibition of TNBC cell apoptosis is an early event that leads to increased tumorigenicity [8]. Thus, it is of great clinical value to identify potential early-induced apoptosis biomarkers to improve the diagnosis and prognostic assessment of TNBC [9].

DNAJB4 (Dnaj heat shock protein family (Hsp40) member B4) is human heat shock protein family (Hsp40) member B4 and belongs to the DnaJ protein family. It is localized on human chromosome 1p31.1, mainly in the mitochondrial matrix, as an auxiliary molecular chaperone of Hsp70 protein [10, 11]. This gene is a strong tumor suppressor in bladder cancer and lung cancer, and its downregulation may serve as a good biomarker for predicting cancer patients’ prognosis [12, 13]. Studies have found that it plays an important role in proliferation, motility, invasion, tumorigenesis, and cell cycle progression [14, 15]. Recently, it has been reported through bioinformatics research that DNAJB4 is involved in multiple signaling pathways in breast cancer and immune function. However, the function of DNAJB4 in TNBC cell apoptosis is still unclear to date [16, 17]. Therefore, further understanding its role in TNBC cell apoptosis is significant for the diagnosis and treatment of diseases.

The Hippo signaling pathway is a signaling pathway that inhibits cell growth [18]. Recent studies have confirmed that the Hippo signaling pathway also plays an important role in cancer cell apoptosis [19]. At the heart of the Hippo pathway is a kinase cascade in which Mst1/2 (the Drosophila Hippo homolog) kinase and SAV1 form a complex that phosphorylates and activates LATS1/2. LATS1/2 kinases in turn phosphorylate and inhibit the transcriptional co-activators YAP and TAZ, two major downstream effector molecules of the Hippo pathway. After dephosphorylation, YAP/TAZ is translocated to the nucleus and interacts with TEAD1-4 and other transcription factors to induce the expression of genes that promote cell proliferation and inhibit apoptosis. After the membrane protein receptor upstream of the Hippo signaling pathway senses the growth inhibitory signal from the extracellular environment, it undergoes a series of kinase phosphorylation reactions, and finally acts on the downstream effectors YAP and TAZ [20, 21].

In this research, DNAJB4 expression was significantly downregulated in TNBC tissues and cell lines. A series of gain- and loss-of-function in vitro and in vivo assays were performed to explore the function of DNAJB4 in TNBC cell apoptosis. Loss of DNAJB4 inhibited TNBC cell apoptosis and promoted tumorigenicity in vitro and in vivo, but overexpression resulted in the opposite. It was investigated how DNAJB4 could regulate the apoptosis of TNBC cells. Loss of DNAJB4 regulated TNBC cell apoptosis with suppression of the Hippo signaling pathway, and the result was reversed after overexpression. Therefore, DNAJB4 may be a prognostic marker and a potential therapeutic target for TNBC.

## Methods

The First Affiliated Hospital of Wannan Medical College's Ethic Committee for Clinical Research has reviewed and approved the current study (IRB file No. 2019070). Written informed consent was obtained from all participants. The experimental research on xenografted tumor model has been approved by the Ethic Committee for Clinical Research of the First Affiliated Hospital of Wannan Medical Collegeand reporting of these experiments complied with the ARRIVE (Animal Research: Reporting of In Vivo Experiments) guidelines. These studies were conducted in accordance with the Declaration of Helsinki (https://www.wma.net/what-we-do/medical-ethics/declaration-of-helsinki/).

### Cell culture

Human normal breast cell (MCF-10A) and breast cancer cell lines (MDA-MB-231, BT-549, MDA-MB-453, MCF-7) were acquired from the Chinese Academy of Sciences Cell Bank (Shanghai, China). The cell lines were characterized by DNA fingerprinting, cell vitality detection, isozyme detection, and mycoplasma detection. The last cell characterization was performed on September 2021. Human normal breast cell MCF-10A was cultured in a special medium from Procell (CM-0525,Procell,China), and the main components are DMEM + 5% Horse Serum + 20 ng/mL EGF + 0.5 µg/mL hydrocortisone + 10 µg/mL insulin + 1% NEAA + 1% P/S. MBA-MD-231 and MDA-MB-453 were cultured in Leibovitz’s L-15 (M210915, CELLCOOK, China) + 10% FBS (SJ06513, LONSA SCIENCE SRL, UY) + 1% P/S (SV30010, Hyclone, USA) medium. BT-549 was cultured in RPMI-1640(8122138, Gibco, USA) + 10% FBS (SJ06513, LONSA SCIENCE SRL, UY) + 1% P/S (SV30010, Hyclone, USA) medium. MCF-7 was cultured in DMEM (Invitrogen, Carlsbad, CA, USA) + 10% FBS (SJ06513, LONSA SCIENCE SRL, UY) + 1%L-alanyl-L-glutamine + 10 µg/mL insulin + 1% NEAA + 1% P/S. All the cells were incubated in 5% CO_2_ at 37 °C.

### Cell transfection

The DNAJB4 knockdown lentivirus was obtained from OBiO (Shanghai, China). Breast cancer cell lines were plated in 6-well dishes at 50% confluence and infected with DNAJB4 knockdown lentivirus (termed as shDNAJB4), or a scramble control (termed as shCtrl) in MDA-MB-231 and BT-549 cells, respectively. Pools of stable transductions were generated by selection using puromycin (4 µg/ml) for 2 weeks. DNAJB4 overexpressed plasmids were obtained from OBiO (Shanghai, China). The cell transfection protocol described above followed the manufacturer's instructions. After transfection, cells were cultured and collected for the following experiments.

### Quantitative real-time polymerase chain reaction (qRT-PCR)

RNA was extracted from cells or tissues using TRIzol reagent (Invitrogen, Burlington, ON, Canada), and 0.4 μg RNA was adopted to synthesize cDNA using a first-strand cDNA synthesis kit (Thermo Fisher Scientific, Waltham, MA, USA). The RNA concentration was examined using the NanoDrop 2000 spectrophotometer (Thermo Fisher Scientific, Waltham, MA, USA). qRT-PCR analysis was performed using the CFX-96 system (BioRad, Hercules, CA, USA) according to the manufacturer’s instructions. PCR amplification was conducted as follows: initial denaturation at 95 °C for 30 s, followed by 40 cycles of 95 °C for 22 s and 60 °C for 30 s. GAPDH were used as controls. The sense primer of DNAJB4 is 5'-CCAGCAGACATTGATTTTTATCATT-3'. The antisense primer of DNAJB4is 5'-CCATCCAGTGTTGGTACATTAATT-3'. The senseprimer of GAPDH is 5′-CCAGGTGGTCTCCTCTGA-3′, and antisense primer of GAPDH is 5′-GCTGTAGCCAAATCGTTGT-3′. Relative expression levels were calculated by the 2^−ΔΔCT^ method. Each sample was repeated in triplicate and analyzed using the Relative Quantification Software.

### Western blot assay

Proteins were extracted from cells or tissues using RIPA lysis buffer (Sigma-Aldrich, St Louis, MO). The concentration of proteins was measured using a BCA assay kit (Bio-Rad Laboratories, Hercules, CA, USA). The 10% SDS-PAGE isolated protein was transferred to a 0.22 μm nitrocellulose (NC) membrane (GE Healthcare, Piscataway, NJ, USA). The membrane was sealed with 5% skim milk at room temperature for 2 h. Then, the membranes were incubated at 4 °C in specific primary antibodies including DNAJB4 (1:1000, PA5-49925, Invitrogen, Burlington, ON, Canada), MST1(1:1000, #14946, Cell Signaling Technology, Danvers, MA, USA), LAST1(1:1000, #3477, Cell Signaling Technology, Danvers, MA, USA), YAP(1:1000, #14074, Cell Signaling Technology, Danvers, MA, USA), p-MST1(1:1000, #49332, Cell Signaling Technology, Danvers, MA, USA), p-LAST1(1:1000, #8654, Cell Signaling Technology, Danvers, MA, USA), p-YAP(1:1000, #13008, Cell Signaling Technology, Danvers, MA, USA), Bax(1:1000, #5023, Cell Signaling Technology, Danvers, MA, USA), Bak(1:1000, #12105, Cell Signaling Technology, Danvers, MA, USA), Bcl-xS(1:1000, #2764, Cell Signaling Technology, Danvers, MA, USA), Cleaved Casepase3(1:1000, #9664, Cell Signaling Technology, Danvers, MA, USA), Bcl-2(1:1000, #15071, Cell Signaling Technology, Danvers, MA, USA), Bax(1:1000, #41162, Cell Signaling Technology, Danvers, MA, USA), Bcl-xl(1:1000, #2764, Cell Signaling Technology, Danvers, MA, USA) and β-Actin (1:3000, A1978, Sigma, Victoria, BC, Canada) at 4 °C overnight. The membranes were washed with 0.1% TBST three times for 5 min each, incubated with anti-mouse or anti-rabbit horseradish peroxidase-conjugated secondary antibody (Cell Signaling Technology, Danvers, MA, USA) for 2 h, and then washed again with 0.1% TBST three times for 5 min each. Chemiluminescent ECL Plus reagents (Pierce, USA) were added to visualize the reaction products. The membranes were scanned with Tanon 5200 (Tanon, Shanghai, PR China). The band intensity was measured by densitometry using the Quantity One Software (Tanon, Shanghai, PR China). The protein levels were normalized with that of β-actin. All experiments were repeated in triplicate, and the representative results were shown.

### Detection of apoptosis by flow cytometry

Cells (MDA-MB-231, BT-549) were cultured in a 6-well plate, and when the cell growth reached 60%-70%, the old medium was aspirated and dealt with according to the experimental needs, and the culture was continued. According to the experimental processing time, the cell culture medium was aspirated into a suitable centrifuge tube, the adherent cells were washed once with PBS, and an appropriate amount of trypsin cell digestion solution was added to digest the cells. Incubation was performed at room temperature until the adherent cells were removed by gentle pipetting, and then the trypsinized cell digestion solution was aspirated. Over-digestion with trypsin was avoided. It should be noted that suspension cells do not need trypsinization, as they can be directly collected into centrifuge tubes. The cell culture solution collected in step (2) was added, mixed slightly, transferred to a centrifuge tube, and centrifuged at 1000 g for 5 min. Then, the supernatant was discarded, and the cells were collected, gently resuspended with PBS, and counted. Of note, adding the cell culture medium in step (2) can collect suspended apoptotic or necrotic cells. On the other hand, the serum in the cell culture medium can effectively inhibit or neutralize the residual trypsin, and enzymes will digest and degrade subsequently added Annexin V-FITC, resulting in failure of staining.

Further, 50,000–100,000 resuspended cells were taken, centrifuged at 200 g for 5 min, and the supernatant was discarded. Then, 195 μl Annexin V-FITC binding solution was added to gently resuspend the cells. An additional 5 μl Annexin V-FITC was added and mixed gently, followed by incubation at room temperature (20–25 °C) for 10 min in the dark. Aluminum foil was used to protect from light. Then, the cells were centrifuged at 200 g for 5 min, the supernatant was discarded, and 190 μl Annexin V-FITC binding solution was added to resuspend the cells. The cells were mixed gently and placed in an ice bath to avoid light. For flow cytometry detection, Annexin V-FITC shows green fluorescence, and PI shows red fluorescence.

### Xenografted tumor model

Six weeks old (average weight: 15 g) BALB/c nude mice (male/female ratio 1:1) were obtained from the Shanghai Institute of Material Medical, Chinese Academy of Science, and maintained under specific pathogen-free conditions. No statistical method was applied for the sample size estimation of the animal study. Five nude mice were enrolled in each experimental group in an unrandomized manner to ensure the precision of the results. The experimental protocol was approved by the Wannan Medical College Animal Experimental Ethics Committee and reporting of these experiments complied with the ARRIVE (Animal Research: Reporting of In Vivo Experiments) guidelines. The cells (2 × 10^6^ MDA-MB-231-shDNAJB4 cells and MDA-MB-231-shCtrl cells) were injected subcutaneously into the right dorsal flank. The tumor sizes were measured daily using a Vernier caliper when the tumors were readily visible. Tumor formation in nude mice was observed by measuring the tumor volume calculated by the following formula: volume = (length × width^2)/2. On day 35, animals were euthanized, and tumors were excised and weighed. The exclusion criterion of the animal experiments was that the bodyweight of the mouse was significantly changed compared to the others. The xenograft tumor was seriously decomposed, which influenced the measurement of the tumor volume.

### Immunohistochemical (IHC) staining

The paraffin-embedded mouse tumor tissues were cut into 5 μm thick slices for immunohistochemical analysis of Ki67 and Caspase3 proteins. After incubation at 60 °C for 2 h, the tissues were immersed in xylene for deaffinity and ethanol to reduce the rehydration concentration. Then, the sections were incubated with the primary antibodies: Ki67 (1:200, ab16667, Abcam, Cambridge, UK) and Cleaved Caspase3 (1:2000, #9664, Cell Signaling Technology, Danvers, MA, USA). Tissue sections were washed with PBS and the following day incubated with biotinylated secondary antibodies for 2 h at room temperature. Finally, tissue sections were processed using the immunompure-metal enhanced DAB substrate kit according to the manufacturer's instructions.

### Rescue experiments

Rescue experiments were performed in MDA-MB-231 and BT-549 cells with silencing DNAJB4, and MDA-MB-231 cell lines overexpressing DNAJB4. MDA-MB-231 and BT-549 cells were first stably transfected with negative control shRNA or DNAJB4 shRNA, and then DNAJB4 overexpression was induced by a plasmid. In addition, DNAJB4 overexpression was induced in MDA-MB-231 cell lines, which were then inhibited with XMU-MP-1 to observe the expression of MST1/LAST1/YAP and p-MST1/p-LAST1/YAP. The rescue experiments of the overexpression group were divided into three groups: negative control shRNA group, DNAJB4 shRNA group, and DNAJB4 shRNA/DNAJB4 cDNA. The rescue experiments of the silencing group were divided into three groups: control cDNA group, DNAJB4 cDNA group, and DNAJB4 cDNA/XMU-MP-1 group. The following assays were performed by western blot.

### Bioinformatics analysis

The expression of DNAJB4 and breast cancer clinical data were down loaded from Gene Expression Profiling Interactive Analysis (GEPIA) database (https: http://gepia2.cancer-pku.cn/). Cut-off values were determined using mean expression level of breast cancer.

### Statistical analysis

All data from three independent experiments were expressed as mean ± SEM. Statistical analyses using SPSS 26.0 software (SPSS Inc., USA). Student’s t-test was used to examine the differences between two groups. And differences between multiple groups were analyzed by one-way ANOVA. The difference was statistically significant when P < 0.05.

## Results

### DNAJB4 expression is highly downregulated in TNBC cells and tissues

**Fig. 1:**
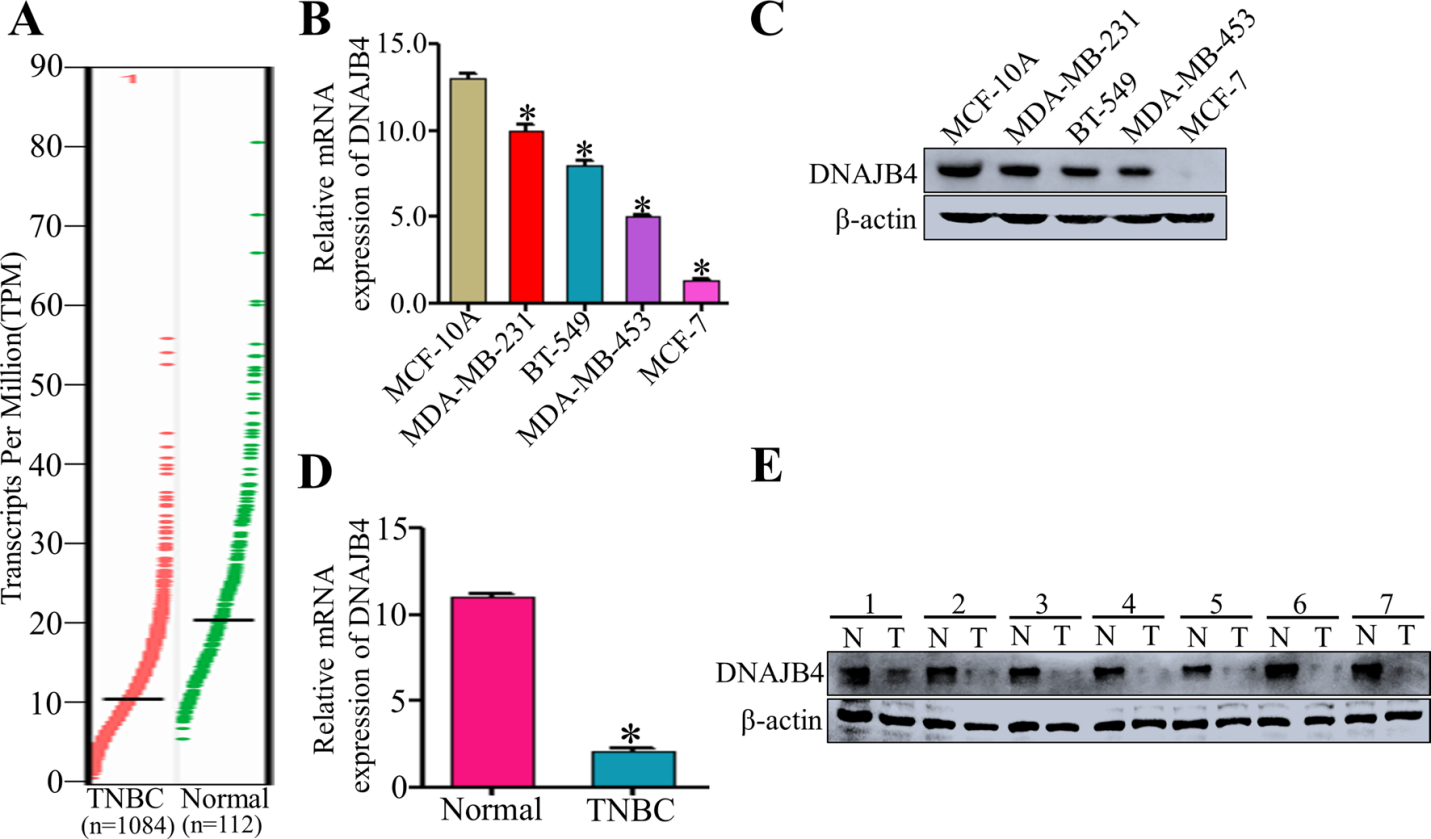
DNAJB4 expression is highly downregulated in breast cancer cells and tissues. **A** Relative DNAJB4 mRNA expression in normal breast tissues (n = 112) and breast cancer tissues (n = 1084) acquired from the GEPIA database. Bar graph data are presented as the mean ± SEM; *, P < 0.05. **B** Relative DNAJB4 mRNA expression in normal breast cell (MCF-10A) and breast cancer cell lines (MDA-MB-231, BT-549, MDA-MB-453, and MCF-7) determined by qRT-PCR. Bar graph data are presented as the mean ± SEM; *, P < 0.05. **C** Western blot analysis of DNAJB4 expression in normal breast cell (MCF-10A) and breast cancer cell lines (MDA-MB-231, BT-549, MDA-MB-453, and MCF-7). β-actin was used as a loading control. **D** Relative DNAJB4 mRNA expression in matched primaryTNBC tissues and adjacent noncancerous tissues determined by qRT-PCR. Bar graph data are presented as the mean ± SEM; *, P < 0.05. **E** Western blot analysis of DNAJB4 expression in seven pairs of matched primary TNBC tissues (T) and adjacent noncancerous tissues (ANT). β-actin was used as a loading control

The Gene Expression Profiling Interactive Analysis (GEPIA) database showed that the DNAJB4 expression was significantly downregulated in breast cancer tissues than normal breast tissues (P < 0.05) (Fig. 1A). The DNAJB4 expression in a series of breast cancer cell lines and the normal breast cell was measured by qRT-PCR and western blot analyses. The results showed that the DNAJB4 expression was markedly downregulated in the MDA-MB-231, BT-549, MDA-MB-453, and MCF-7 cell lines compared to normal breast cells (MCF-10A) (P < 0.05) (Fig. 1B–C). Furthermore, the qRT-PCR and western blot analyses revealed that the expression levels of DNAJB4 in the seven pairs of human **TNBC** tissues were lower compared to the matched adjacent noncancerous tissues, which confirmed the downregulation of DNAJB4 in **TNBC** tissue (Fig. 1D–E). Taken together, the above results suggested that the DNAJB4 expression was highly downregulated in **TNBC**.

### DNAJB4 knockdown inhibits TNBC cell apoptosis in vitro

**Fig. 2:**
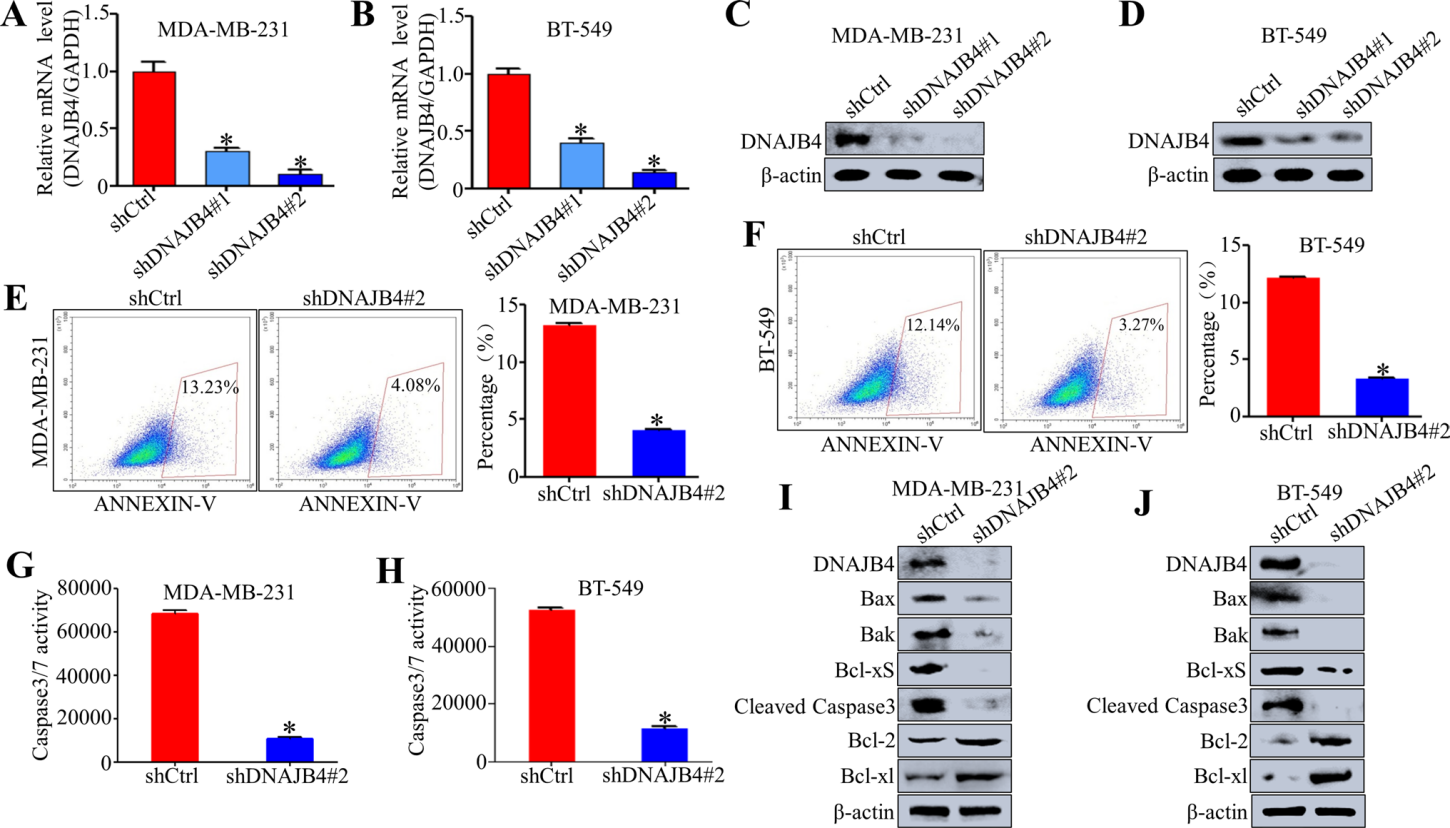
Knockdown of DNAJB4 inhibits TNBC cell apoptosis in vitro. **A**, **B** Relative DNAJB4 mRNA expression in MDA-MB-231 and BT-549 cells expressing shDNAJB4 #1 and shDNAJB4 #2 analyzed by qRT-PCR. Experiments were conducted in triplicate. Bar graph data are presented as the mean ± SEM; *, P < 0.05. **C**, **D** Western blot analysis of DNAJB4 expression in DNAJB4-silenced MDA-MB-231 and DNAJB4-silenced BT-549 cells. β-actin was used as a loading control. **E**, **F** Flow cytometry assay was used to detect the effect of cell apoptosis in DNAJB4-silenced MDA-MB-231 and DNAJB4-silenced BT-549 cells. Representative profiles are shown on the left, and the percentage of cells was statistically analyzed on the right. Experiments were conducted in triplicate. Bar graph data are presented as the mean ± SEM; *, P < 0.05. **G**, **H** The apoptosis level of DNAJB4-silenced MDA-MB-231 and DNAJB4-silenced BT-549 cells were detected by Caspase3/7 activity kit. Experiments were conducted in triplicate. Bar graph data are presented as the mean ± SEM; *, P < 0.05. **I**, **J** Western blot analysis of the expression level of crucial cell apoptosis regulatory proteins in DNAJB4-silenced MDA-MB-231 and DNAJB4-silenced BT-549 cells. β-actin was used as loading control

To explore the functional role of DNAJB4 in breast cancer cells, we stably knocked down the expression of DNAJB4 via two DNAJB4-specific lentiviral shRNAs (shDNAJB4 #1 and shDNAJB4 #2) in MDA-MB-231 and BT-549 cells. As shown in Fig. 2A–D, shDNAJB4#2 exhibited the most evident knockdown efficiency and was chosen for the subsequent in vivo and mechanism experiment. After DNAJB4 was knocked down in the MDA-MB-231 and BT-549 breast cancer cells, the apoptosis of breast cancer cells was detected by flow cytometry. It was found that the apoptosis rate of the MDA-MB-231 breast cancer cells decreased after DNAJB4 knockdown, as well as that of the BT-549 cells (Fig. 2E–F). Moreover, Caspase3/7 activity was also detected in DNAJB4-silenced MDA-MB-231 and BT-549 cells, which confirmed that DNAJB4 knockdown inhibited the breast cancer cell apoptosis (Fig. 2G–H). Additionally, DNAJB4 knockdown enhanced the expression levels of the anti-apoptotic proteins Bcl-2 and Bcl-xl, and decreased the levels of the pro-apoptotic proteins Bax, Bak, Bcl-xS, and Cleaved Caspase3 in MDA-MB-231 and BT-549 cells (Fig. 2I–J). Therefore, the above results indicate that knockdown of DNAJB4 can inhibit the apoptosis of TNBC cells in vitro.

### DNAJB4 overexpression promotes TNBC cell apoptosis in vitro

**Fig. 3:**
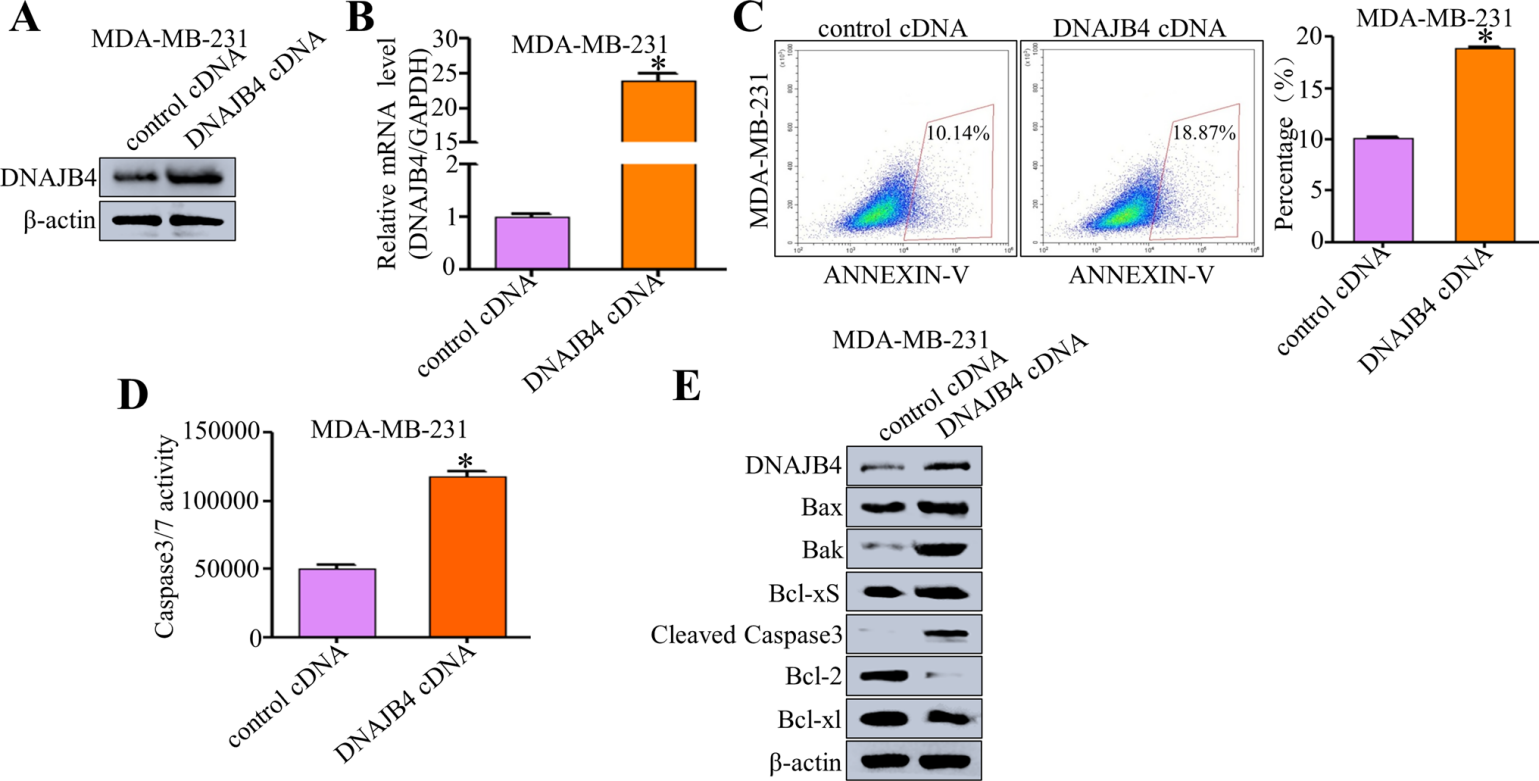
DNAJB4 overexpression promotes TNBC cell apoptosis in vitro*.* **A** Relative DNAJB4 mRNA expression in MDA-MB-231 cells expressing DNAJB4cDNA analyzed by qRT-PCR. Experiments were conducted in triplicate. Bar graph data are presented as the mean ± SEM; *, P < 0.05. **B** Western blot analysis of DNAJB4 expression in DNAJB4-overexpressed MDA-MB-231 cells. β-actin was used as a loading control. **C** Flow cytometry assay was used to detect the effect of cell apoptosis in DNAJB4-overexpressed MDA-MB-231 cells. Representative profiles are shown on the left, and the percentage of cells was statistically analyzed on the right. Experiments were conducted in triplicate. Bar graph data are presented as the mean ± SEM; *, P < 0.05. **D** The apoptosis level of DNAJB4-overexpressed MDA-MB-231 cells were detected by Caspase3/7 activity kit. Experiments were conducted in triplicate. Bar graph data are presented as the mean ± SEM; *, P < 0.05. **E** Western blot analysis of the expression level of crucial cell apoptosis regulatory proteins in DNAJB4-overexpressed MDA-MB-231 cells. β-actin was used as a loading control

To further explore the biological role of DNAJB4 overexpression in breast cancer, we used an overexpression plasmid to stably elevate DNAJB4 expression. After DNAJB4 overexpression in MDA-MB-231 breast cancer cells, the efficiency of DNAJB4 overexpression was detected by qRT-PCR and western blot (Fig. 3A–B). The results showed that the DNAJB4 overexpression efficiency of the plasmid was good, which was used for subsequent functional mechanism experiments. After overexpression of DNAJB4 in MDA-MB-231 breast cancer cells, the apoptosis rate of the breast cancer cells was detected by flow cytometry and was found to be increased (Fig. 3C). Meanwhile, after overexpression of DNAJB4 in MDA-MB-231 breast cancer cells, the apoptosis of cells was detected by Caspase3/7 activity kit. The results showed that the Caspase3/7 activity of MDA-MB-231 breast cancer cells was increased (Fig. 3D). Furthermore, the expression of apoptosis-related proteins was detected by western blot. It was found that the expression of pro-apoptotic proteins (Bax, Bak, Bcl-xS, and Cleaved Caspase3) was increased, and the expression of anti-apoptotic proteins (Bcl-2 and Bcl-xl) was decreased (Fig. 3E). These findings indicated that DNAJB4 overexpression promoted TNBC cell apoptosis in vitro.

### DNAJB4 knockdown promotes the tumorigenicity of TNBC cells in vivo

**Fig. 4:**
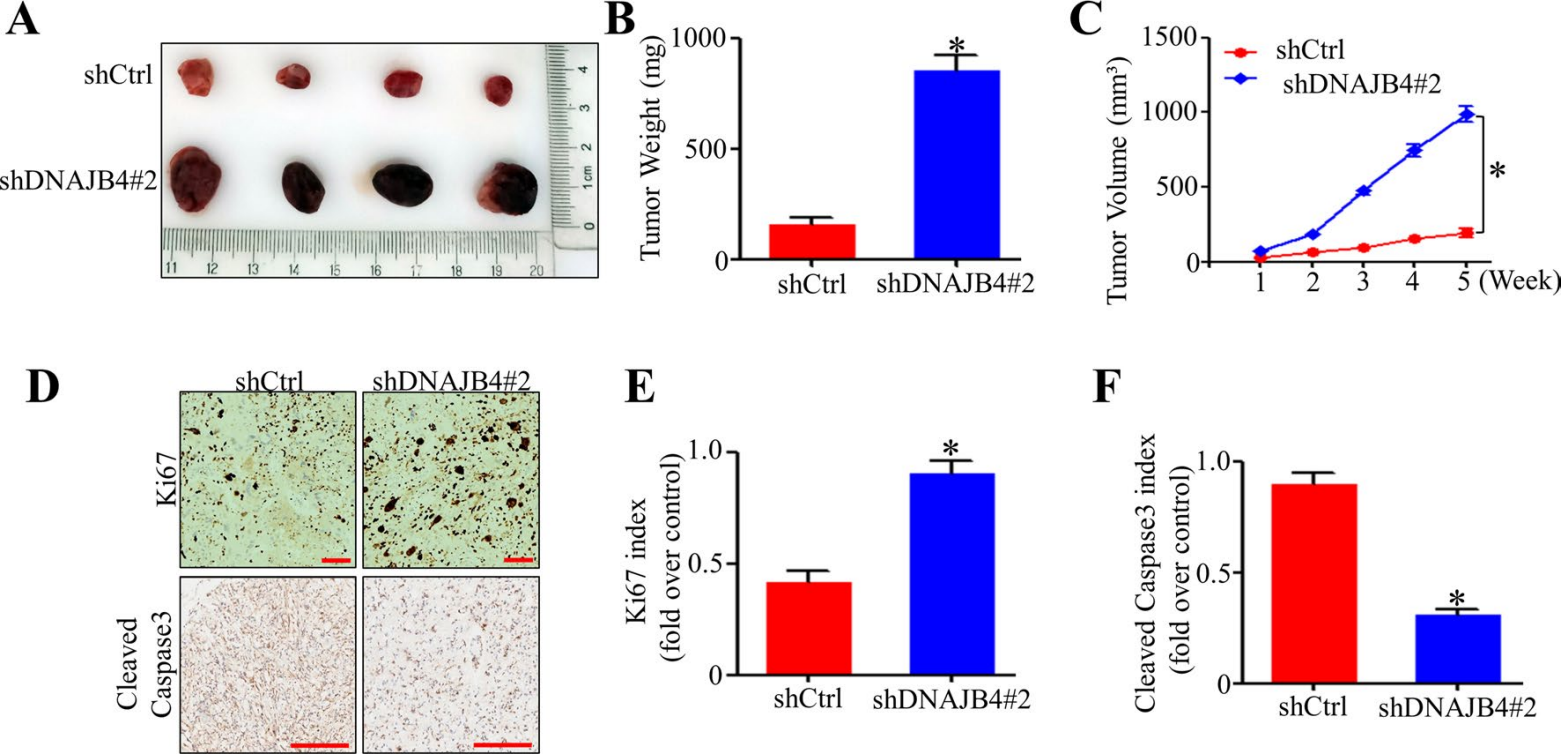
DNAJB4 knockdown promotes the tumorigenicity of TNBC cells in vivo*.* **A** Representative images of the corresponding xenografts in the shCtrl and shDNAJB4 groups (n = 4). **B** The weight of tumors in the shCtrl and shDNAJB4 groups. Bar graph data are presented as the mean ± SEM; *, P < 0.05. **C** The growth curve of the tumors in the shCtrl and shDNAJB4 groups. Bar graph data are presented as the mean ± SEM; *, P < 0.05. **D**–**F** The Ki67 and Cleaved Caspase3 expression levels were determined in xenograft model tumor tissues by immunohistochemistry. All data are presented as the mean ± SEM; *, P < 0.05. Scale bars, 100 μm (200 × magnification) for panels

A mouse xenograft model was established to determine whether DNAJB4 could influence tumorigenesis in vivo. The shRNA or DNAJB4 shRNA MDA-MB-231 cells were injected at the flank into nude mice. After 5 weeks of injection, the volume and weight of the shDNAJB4 group were significantly increased compared to those of the shCtrl group (Fig. 4A–C). In addition, the Ki67 and Cleaved Caspase3 expression levels were upregulated in the tumors formed by DNAJB4-downexpressing breast cancer cells compared to DNAJB4 normal-expressing cells (Fig. 4D–F). These results demonstrated that DNAJB4 knockdown is crucial in promoting TNBC tumorigenesis.

### DNAJB4 regulates TNBC cell apoptosis via the Hippo signaling pathways

**Fig. 5:**
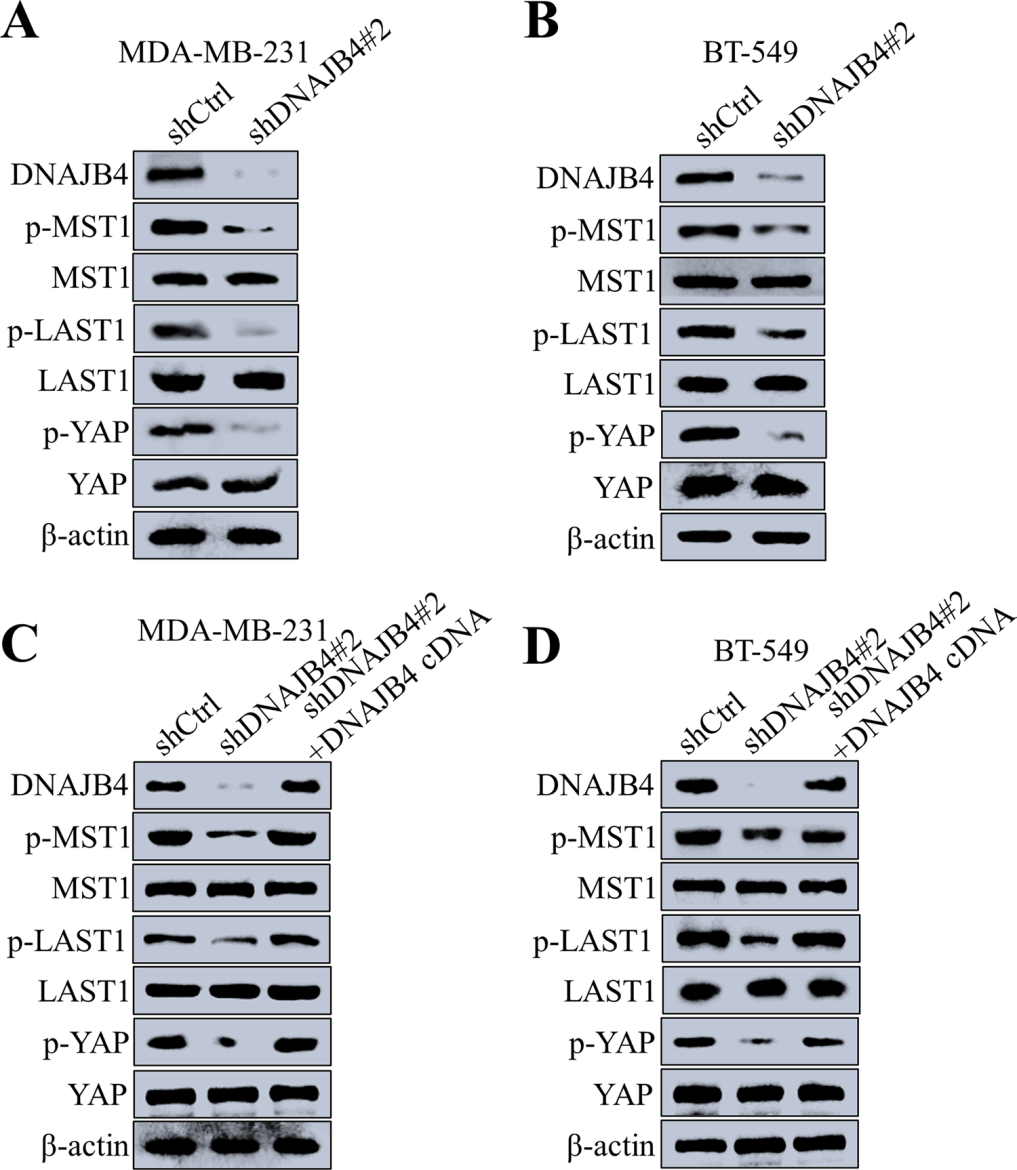
DNAJB4 knockdown attenuates TNBC cell apoptosis through suppression of the Hippo signaling pathway. **A**, **B** Changes of Hippo signaling pathway-related proteins after DNAJB4 knockdown in MDA-MB-231 and BT-549 cell, determined by western blot. β-actin was used as a loading control. **C**, **D** Changes of Hippo signaling pathway-related proteins in DNAJB4 knockdown MDA-MB-231 and BT-549 cells after the addition of cDNA lentivirus overexpressing DNAJB4, determined by western blot. β-actin was used as a loading control

**Fig. 6:**
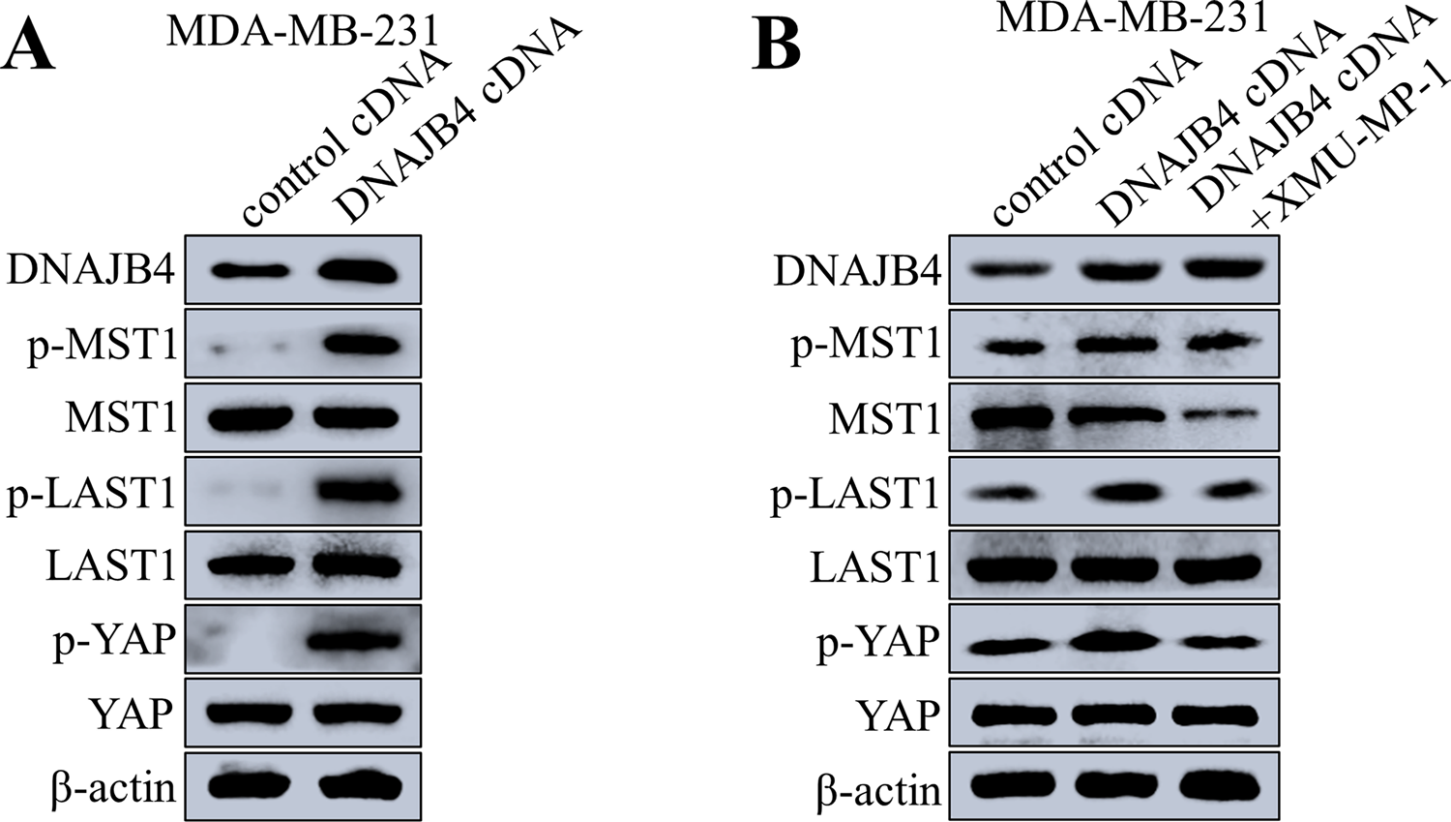
DNAJB4 promotes TNBC cell apoptosis via activating the Hippo signaling pathways. **A** Changes of Hippo signaling pathway-related proteins after DNAJB4 overexpression in MDA-MB-231 cell, determined by western blot. β-actin was used as a loading control. **B** Changes of Hippo signaling pathway-related proteins in DNAJB4 overexpressed MDA-MB-231 cells after the addition of XMU-MP-1, an inhibitor of MST1, determined by western blot. β-actin was used as a loading control

After DNAJB4 was knocked down in TNBC cells by MDA-MB-231 and BT-549, the expression of Hippo signaling pathway-related proteins was detected by Western blot. The results showed that the phosphorylation levels of MST1, LATS1, and YAP1 were decreased, and the total protein expression of MST1, LATS1, and YAP1 was unchanged (Fig. 5A–B). After DNAJB4 knock down in MDA-MB-231 and BT-549 TNBC cells, the cDNA lentivirus overexpressing DNAJB4 was added, and the expression of Hippo signaling pathway-related proteins was detected by Western blot. The results showed that the phosphorylation levels of MST1, LATS1, and YAP were restored, and the total protein expression of MST1, LATS1, and YAP1 remained unchanged (Fig. 5C–D). In addition, MDA-MB-231 cell line was transfected with lentivirus overexpressing DNAJB4, and the expression of Hippo signaling pathway-related proteins was detected by Western blot. The results showed that after DNAJB4 overexpression, the phosphorylation levels of MST1, LATS1, and YAP increased, and the total protein expression of MST1, LATS1, and YAP remained unchanged (Fig. 6A). XMU-MP-1, an inhibitor of MST1, was added to MDA-MB-231 cells overexpressing DNAJB4, and western blot was used to detect the expression of Hippo signaling pathway-related proteins. The results showed that the phosphorylation levels of MST1, LATS1, and YAP were restored (Fig. 6B). Therefore, DNAJB4 promotes TNBC cell apoptosis by activating the Hippo signaling pathway.

## Discussion

According to the IARC statistics 2020, breast cancer (BC) ranks first among malignant tumors and fifth in terms of mortality rate worldwide [1]. According to the 2022 statistics from the National Cancer Center, breast cancer (BC) has the highest incidence among Chinese women and accounts for 71,700 deaths, ranking fifth in terms of mortality rate among all cancers. This shows that the burden of malignant tumors is increasing daily, and the situation of cancer prevention and control is poor. Thus, more attention should be paid to these people [22]. At present, anti-her-2 therapy and anti-HR + therapy have significantly improved the prognosis of HER-2 overexpression and HR + breast cancer, while triple-negative breast cancer (TNBC) lacks therapeutic targets. It is very important to identify ways of improving the survival of this population of patients [23]. Therefore, the potential molecular mechanism of TNBC treatment needs to be further explored.

This study provides new insights and strong evidence that DNAJB4 plays a critical role in the tumorigenicity and progression of TNBC. DNAJB4 knockdown decreased the apoptosis of TNBC cells. The loss of DNAJB4 attenuated TNBC cell apoptosis by suppressing the Hippo signaling pathway. Overexpression of DNAJB4 was shown to promote TNBC cell apoptosis by activating the Hippo signaling pathway.

DNAJB4 (Dnaj heat shock protein family (Hsp40) member B4) is a human heat shock protein family (Hsp40) member B4, which was isolated from human liver tissue in 1998. DNAJB4 belongs to the DnaJ protein family and is located on human chromosome 1p31.1, mainly in the mitochondrial matrix, and functions as an auxiliary molecular chaperone of Hsp70 protein [10, 11]. Studies have shown that DNAJB4 can interact with some intracellular proteins and participate in the regulation of tumor occurrence and development through molecular signaling pathways and other factors, but its mechanisms are still unknown [14, 15].

Recent research suggests that this binding to DNAJB4-Src may block Src. Interaction with its partners alters the Src signaling pathway downstream targets in tumor cells, thereby inhibiting tumorigenesis. The formation of the DNAJB4-Src complex inhibits the interaction between Src and its activators, such as FAK, which is responsible for the most critical events in cancer progression [24, 25]. DNAJB4 is a tumor suppressor gene, that has been reported in our previous studies and was discovered through bioinformatics studies. It is involved in multiple signaling pathways in breast cancer and immune function, but many of its functions are still unknown [16].

In our previous study, we evaluated the expression level of DNAJB4 in breast cancer and its clinical significance through a large number of clinical tissue samples. The expression levels of DNAJB4 mRNA and protein in 80 breast cancer tissue samples were detected by qRT-PCR, western blot and IHC, respectively. The results showed that aberrantly low expression levels of DNAJB4 were likely associated with adverse prognoses in breast cancer [16]. In our study, DNAJB4 expression was detected in two TNBC cell lines (MDA-MB-231 and BT-549). Then, the lentiviral vector was constructed to stably knock out DNAJB4 to elucidate the biological behavior of DNAJB4 in TNBC, and stably transduced MDAMB-231 and BT-549 cell lines. The results demonstrated that DNAJB4 knockdown could suppress the apoptosis of MDA-MB-231 and BT-549 in vitro. Meanwhile, overexpression of DNAJB4 in MDA-MB-231 cell line promoted tumor cell apoptosis in vitro. However, the mechanisms of DNAJB4 in TNBC cell apoptosis have not been studied until now.

Aberrations in multiple cellular signaling pathways can regulate tumor development, growth, proliferation, metastasis, and apoptosis [26, 27]. The fundamental cellular signaling cascade involved in these processes is the Hippo signaling pathway [28]. It is an important and well-studied intracellular signaling pathway in tumorigenesis and apoptosis [29]. Various activating mutations in oncogenes and inactivation of tumor suppressor genes have been found in a variety of malignancies with almost all pathway members. The Hippo signaling pathway serves as a target for cancer therapy [30, 31]. In this study, DNAJB4 knockdown could inhibit the Hippo signaling pathway, as the phosphorylation levels of MST1, LATS1, and YAP decreased, while the total protein expression of MST1, LATS1, and YAP remained unchanged. When DNAJB4 was knocked down and then overexpressed in MDA-MB-231 and BT-549 breast cancer cells, Western blot analysis showed that the phosphorylation levels of MST1, LATS1, and YAP were restored, while the total protein expression of MST1, LATS1, and YAP1 remained unchanged. Moreover, the transfection of MDA-MB-231 cell line with lentivirus overexpressing DNAJB4 could increase the phosphorylation levels of MST1, LATS1, and YAP, whereas the total protein expression of MST1, LATS1, and YAP remained unchanged. XMU-MP-1, an inhibitor of MST1, was added to MDA-MB-231 cells overexpressing DNAJB4, resulting in the restoration of the phosphorylation levels of MST1, LATS1, and YAP.

In conclusion, the current study demonstrated that DNAJB4 was highly decreased in TNBC patient samples and cell lines. Loss or overexpression of DNAJB4 attenuated or enhanced breast cancer cell apoptosis through the Hippo signaling pathway. Full understanding of the exact role of DNAJB4 in TNBC may provide an opportunity to develop a novel therapeutic strategy by inhibiting its expression in TNBC cells. Translational studies on the clinical application of DNAJB4 are required to develop a methodology and evaluate the molecular diagnostic application of DNAJB4 in TNBC.

## Supplementary Information

Additional file1 (TIF 142 KB)

## Data Availability

The datasets used and/or analyzed during the current study are available from the corresponding authors on reasonable request.
