# Supplementary figures and images for: DNAJB4 promotes triple-negative breast cancer cell apoptosis via activation of the Hippo signaling pathway
Source: Discov Oncol. 2023 Apr 4;14:40. doi: 10.1007/s12672-023-00645-y (PMC10070573; doi:10.1007/s12672-023-00645-y)

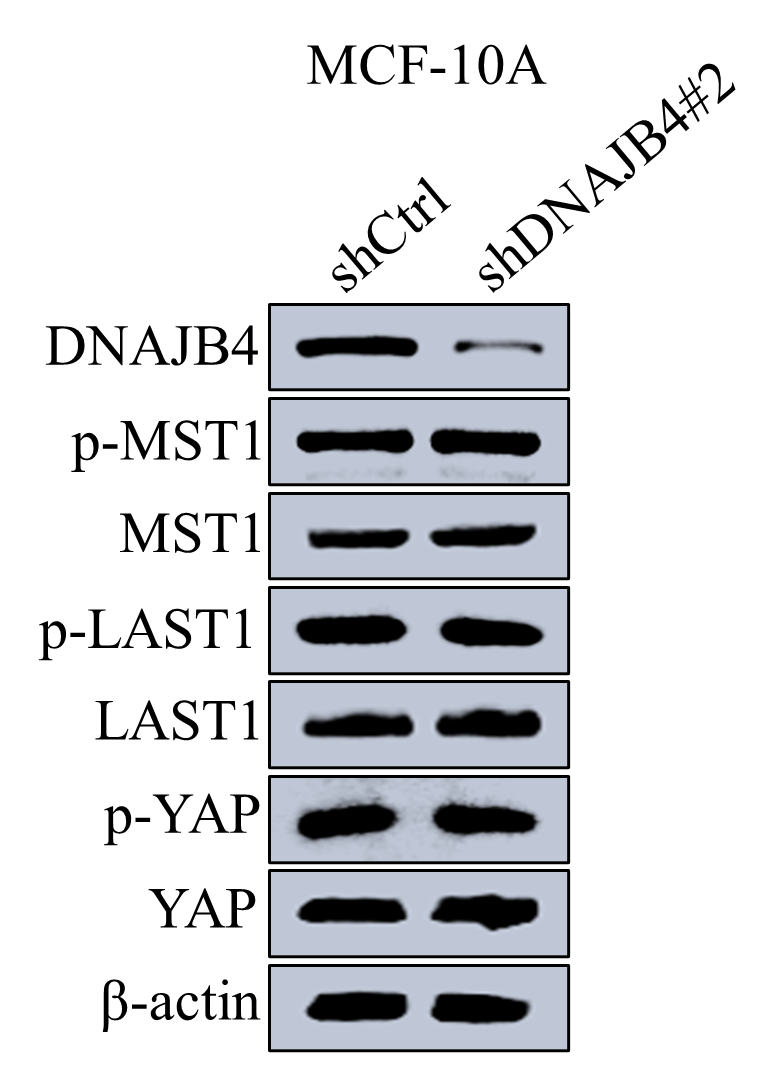

Supplement: Supplementary file 1 — Additional file1 (TIF 142 KB) [file 12672_2023_645_MOESM1_ESM.tif]
